# Artificial Intelligence and Health Inequities in Dietary Interventions on Atherosclerosis: A Narrative Review

**DOI:** 10.3390/nu16162601

**Published:** 2024-08-07

**Authors:** Dominique J. Monlezun, Keir MacKay

**Affiliations:** 1 Department of Hospital Internal Medicine, Mayo Clinic, Rochester, MN 55905, USA; mackay.keir@mayo.edu; 2 Department of Cardiology, The University of Texas MD Anderson Cancer Center, Houston, TX 77030, USA; 3 Faculty of Bioethics, Ateneo Pontificio Regina Apostolorum, 00163 Rome, Italy; 4 School of Bioethics, Universidad Anahuac México, Mexico City 52786, Mexico; 5 Center for Artificial Intelligence and Health Equities, Global System Analytics & Structures, Rochester, MN 55905, USA

**Keywords:** nutrition, diet, atherosclerosis, artificial intelligence, health inequities

## Abstract

Poor diet is the top modifiable mortality risk factor globally, accounting for 11 million deaths annually with half being from diet-linked atherosclerotic cardiovascular disease (ASCVD). Yet, most of the world cannot afford a healthy diet—as the hidden costs of the inadequate global food system total over USD 13 trillion annually—let alone the much more clinically, financially, and ecologically costly and resource-intensive medical interventions required to address the disease progression and acute complications of ASCVD. Yet, AI is increasingly understood as a force multiplying revolutionary technology which may catalyze multi-sector efforts in medicine and public health to better address these significant health challenges. This novel narrative review seeks to provide the first known overview of the state-of-the-art in clinical interventions and public health policies in healthy diets for ASCVD, accelerated by health equity-focused AI. It is written from the first-hand practitioner perspective to provide greater relevance and applicability for health professionals and data scientists. The review summarizes the emerging trends and leading use cases in population health risk stratification and precision public health, AI democratizing clinical diagnosis, digital twins in precision nutrition, and AI-enabled culinary medicine as medical education and treatment. This review may, therefore, help inform and advance the evidence-based foundation for more clinically effective, financially efficient, and societally equitable dietary and nutrition interventions for ASCVD.

## 1. Introduction

Poor diet is the top modifiable mortality risk factor globally [1]. It accounts for 11 million deaths annually with half being from diet-linked atherosclerotic cardiovascular disease (ASCVD), which is the top clinical cause of death [1]. Excessive fat (particularly trans and saturated fatty acids), salt, and sugar intake increase fatty deposits in the inner arterial lining. These can eventually produce peripheral arterial disease (with resultant limb loss), chronic kidney disease, cerebrovascular accidents (or strokes), and ASCVD depending on the location and severity of that pathological build up that excessively limits sufficient blood flow. The daily average cost for a healthy diet internationally is $3.66, significantly higher than the USD 2.15 poverty line [2]. This means that approximately 42% of the global human community cannot afford the diet their health requires. Further, the United Nations (UN) in 2023 estimated that the hidden costs of our world’s insufficient food system totaled nearly USD 13 trillion annually—10% of global gross domestic product (GDP) overall and nearly 30% in low-income countries—with the largest source at 73% coming from diet-linked diseases [3]. The hidden costs refer to the net negative impact of a product or service not reflected in its market price. These health and financial costs of poor diets are only exacerbated by the inter-connected debt, climate, and conflict polycrises increasingly plaguing communities globally, and disproportionately impacting low- and middle-income countries or LMICs. Populations which become sicker become less productive, poorer, and more prone to political instability and conflict which in turn feeds back into this disease–poverty ‘doom loop’ [4]. Amid this societal context, the explosive 2023 launch of ChatGPT catalyzed a global awareness of how artificial intelligence (AI) is progressively unleashing an unprecedented scale, scope, and speed of the diffusion of this accessible, affordable, and adaptable revolutionary technology. This trend facilitates and is furthered by the increasing digitalization of the global health ecosystem [5]. This includes the growing use of AI for health, operationalized as the UN’s Sustainable Development Goals (SDGs) that emphasize sufficient healthy diet and its relation to poverty reduction and health maximization in its top three goals [5]. Similarly, the American Heart Association (AHA) and American College of Cardiology (ACC) recognize the well-established link between diet and ASCVD (and the management of the related conditions of obesity, diabetes, and hypertension). They recommended in their 2019 guidelines a healthy diet for the primary prevention of ASCVD (mirroring the consensus of at least 10 other professional associations), including increasing the consumption of vegetables, fruits, nuts, whole grains, fish, and lean protein while reducing the consumption of trans fats, cholesterol, red meats, sweetened beverages, and refined carbohydrates. These recommendations also include nutrition screening in clinical visits and consultations with registered dieticians (RDs) as indicated.

An extensive and multi-decade body of scientific literature supports these recommendations and the above biological mechanisms underlying them. But the growing prevalence of poor diet-linked ASCVD underlines the unique and force-multiplying benefit AI may provide to more rapidly design, optimize, and scale affordable, equitable, and sustainable health interventions to reverse this worsening and worrying trend. However, there are no published empirical research studies on AI, diet, and ACSDVD (nor on the related health inequities). This research gap undermines the possibility to conduct a sufficiently robust, informative, and valid systematic review identifying the state-of-the-art on this novel intersection topic. We, therefore, sought to provide the first known health equity-focused narrative review of AI-accelerated clinical interventions and public health policies in healthy diets for ASCVD. An additional novel aspect of it is that it is performed by a triple doctorate-trained practicing physician–data scientist and AI ethicist (DJM) to provide a first-hand perspective of the leading use cases in this topic by which the review is organized for a more relevant and convenient personalization of those use cases for diverse communities’ local needs globally.

## 2. Population Health Risk Stratification and Precision Public Health

The identification of at-risk individuals and populations informs the first step of designing dietary interventions and policies which are meant to serve them. A 2024 *Nutrients* study of the 1999–2018 National Health and Nutrition Examination Survey (NHANES) used a machine learning type of AI called extreme gradient boosting with a Cox proportional hazards model to advance this step [6]. Boosting generally seeks to solve supervised learning problems with decision trees, meaning that the AI model identifies patterns in inputs or variables from labeled datasets to predict outputs or outcomes in a way that can represented as a tree with each input and output signified as a ‘leaf’. The study showed a significant association between age-specific dietary patterns and metabolic syndrome incidence. The study especially highlighted the differential impact of cholesterol, theobromine, and caffeine on metabolic syndrome risk by specific age groups. It also generated a high-accuracy predictive model identifying individuals at higher metabolic syndrome risk which can in turn enhance precision public health through more tailored dietary interventions, and the related potential cost savings through more focused interventions on those most likely to benefit from them. Practically, this means that the AI approach can help better match people according to age and metabolic risk with the optimal dietary pattern to reduce their ultimate ASCVD risk. Such studies are increasingly connecting and complementing medicine and public health, respectively, through the intersection of the former’s population health (focused on a narrower often geographic group served by certain healthcare systems) and the latter’s precision public health (often using AI analyzing Big Data to right-fit timely interventions for the right groups best able to benefit from them regardless of their geography).

As in the case of the above study, such machine learning can guide which age groups may best benefit from targeted dietary interventions for their ASCVD risk. A 2024 *Nature* study demonstrated that geography may additionally be a useful and underexplored dimension of risk stratification and targeted interventions [7]. It used the 2003–2021 CoLaus|PsyCoLaus cohort study dataset to longitudinally show high versus low ASCVD risk clusters across 6203 individuals, primarily driven by body mass index (BMI), and how that risk translates into a greater incidence of eventually developing ASCVD, especially in the setting of obesity. The more complex and complete such AI models become (not just considering people’s age above but also with such clinical information as their BMI and where they live, including in relation to clusters of ASCVD and health resources like clinics and hospitals), the more precisely and individually they can predict ASCVD risk and so inform countermeasures for its prevention, mitigation, and recovery including with optimized diets. Such research builds on the seminal findings from the 2007 *New England Journal of Medicine* research which first showed across 32 years of 12,067 subjects in the Framingham Heart Study how obesity spreads through geographically clustered social networks [8]. We are more likely to eat healthy or unhealthy meals depending on who we are eating with, and whether they have a healthy or obese BMI. A 2024 *Lancet* publication took such findings a step further by showing how generative AI using large language models (LLMs) like in ChatGPT not only enabled a real-time Singapore geographic dashboard of de-identified individual ASCVD risk including with glyated hemoglobin (HbA1c) and low-density lipoprotein-C (LDL-C) [9]. It also allowed the integration of medication and socioeconomic determinants of health data to better tailor preventive cardiology care that can include healthy diet education and support with the long-term tracking of the continuous optimization of this risk stratification, program delivery, and outcome improvements. This generative AI method generally uses artificial neural networks (AANs) within deep learning on huge datasets in a way that supersedes what simple machine learning can do: more complex data informs smarter AI for better predictions. This is because machine learning is a type of supervised learning that requires human programmers to ‘teach’ the AI model what patterns and outcomes to find based on labeling data inputs and outputs, like explicitly telling the model what HbA1c and ASCVD values mean and their relation to each other. Deep learning as unsupervised learning works closer to how our brains do since it means the AI model is fed raw or unlabeled data and minimal instructions to find patterns, leaving it up to the model to increasingly improve its performance through its own trial and error finding the best way to do that pattern recognition and outcome prediction. This particular study, therefore, suggests the practical AI applications of such deep learning to inform which communities served by healthcare systems have the highest ASCVD risk according not just to patients’ individual clinical risks but also their social determinants of health, and thus how to better provide them targeted countermeasures to maximize the impact of limited resources for those most likely to benefit from them. The AI can, for instance, help map out for physicians and public health workers the neighborhoods of family, friend, and neighbor networks with higher rates of diabetes who also have lower income and higher under- or un-employment in a way that can also find novel proxy markers that may be more subtle but earlier warning signs for such eventual higher ASCVD risk (like small increases in diastolic blood pressure), and which may concurrently have less access to healthy food options exacerbating this problem. This can, in turn, guide how the healthcare systems caring for such social networks can partner with public health agencies and grocery stores in these food desert areas to expand services to these communities, and even co-locate satellite clinic locations in or adjacent to those clinics to enhance more affordable, accessible, and full-spectrum health solutions. Understanding not only when but where someone and her/his social networks will develop ASCVD can, therefore, enable more efficient use of health resources across diverse stakeholders in a health ecosystem to prevent, mitigate, and treat. Effective AI can, thus, accelerate managing population health more precisely by translating helpful but overly general maxims (i.e., ‘eat better’) into personalized practical solutions that work in real people’s real-world lives, limitations, and preferences.

## 3. AI Democratizing Clinical Diagnosis

Moving from the population to the individual level, and risk stratification to diagnosis, there are a number of recent AI advances for enhancing the clinical diagnosis of ASCVD to enable improved nutrition interventions. This is particularly important given the worsening global physician shortfall, as over two of every three countries already face shortages, and the World Health Organization projects this will worsen to a shortage of 10 million health workers by 2030—with LMICs bearing the majority of this burden through international brain drain and inadequate domestic education [10]. In response, there is a growing AI-accelerated ‘democratization’ of healthcare through more accessible and affordable diffusion of accurate diagnoses and treatment especially for resource-limited countries and communities. A 2023 *Lancet* study of 7.12 million subjects at over 70 hospitals used an AI technique of ANNs on electrocardiograms (ECGs) without known ASCVD to accurately diagnose obstructive coronary artery disease or CAD (area under the receiver operative curve or AUC of 0.85) and predict acute coronary events at least up to three years out based on them [11]. For comparison, diagnosing obstructive disease in an American cardiac catheterization procedure can be over USD 10,000, while an ECG can be over 10 times cheaper. A 2024 nationally representative study adopted this approach for nutrition by utilizing ANNs across nearly 150 million adult hospitalizations from 2016 to 2020 [12]. It showed that less than 1% of obese patients with CAD receive dietary counseling, and that while diagnosed poor diet increased counseling, CAD actually reduced the odds of receiving dietary counseling by 15%—though reversing such disparities may annually save an additional 88,345 hospitalized lives. Notably, this AI model was developed solely from ICD-10 (International Classification of Diseases) codes, the WHO-standardized classification for diseases used by the majority of countries globally. This suggests that poor diet in ASCVD can be rapidly and automatically diagnosed through a commonly available AI technique, as can the gap between those not receiving dietary counseling for it.

Aside from the technological advances in AI and expanded clinical uses for them, there are expanded geographic applications for them to better democratize this AI including for ASCVD and related dietary risks. A 2024 prospective cohort study from India built on the multi-year partnership between Microsoft’s cloud computing-backed AI Network for Healthcare and Apollo Hospitals as the country’s largest healthcare system [13]. The former empowers the latter with its AI platform delivered remotely according to local hospital and patient needs. The study used a deep learning hazards model across 31,599 adults and nine years to develop its novel ‘Artificial Intelligence-based Risk Score (AICVD)’, with diet being one of the six key included factors, to predict ASCVD events including acute myocardial infarctions with good performance (AUC 0.853). Notably, the score was validated with independent cohorts in India and the Netherlands with superior positive likelihood ratios and accuracy compared to one of the most commonly used scores, the American-based Framingham Heart Risk Score. Not only was more powerful deep learning AI used locally in a LMIC, but it was also used to develop more population-specific risk scores instead of the frequent practice of extrapolating clinical tools globally even when people have significant differences among them that limits the extension of such tools. This process of AI democratization maturing into ‘sovereign AI’ is also manifested by such emerging partnerships as the American company, NVIDIA (Santa Clara, CA, USA), and Reliance Industries (Mumbai, India), India’s largest private corporation, to build India’s own foundation LLM version of ChatGPT [14]. This LLM is being trained in India’s own languages and cultures so it can own its data and the intelligence produced from it for its own prioritized use cases [14]. NVIDIA provides the best-in-class cloud-based AI supercomputing service for Reliance with its own on-site data centers to accelerate its energy-efficient AI applications and services for its half a billion customers. The target capacities for this partnership explicitly focus on such case uses as boosting tele-health services for Indian healthcare systems. This is meant to enhance and expand their care for remote and underserved patients, including by augmenting providers’ more personalized diagnoses and treatment plans for these patients based on their clinical histories, laboratory values, imaging, and acute symptoms. It should be noted that despite the significant upside of such clinical AI democratization including improved efficiencies and equities, there are notable risks and ethical challenges [4]. These include greater data security risks as more individual data are digitalized and accessible to such AI programs and partnerships, how the ownership of the data and its intelligence are shared and disagreements resolved, and how transparent and accountable the process is of this AI development and deployment which is influenced inevitably by the geopolitical and corporate competition producing these technologies. There are, therefore, growing multi-sector, multi-lateral, and multicultural approaches to institutionalize compliance-by-design into the AI’s workforce education in such ethics and its very R&D cycle as companies particularly in the United States and Europe build their products and services to adhere to such regulatory standards as the European Union’s AI Act and America’s Health Insurance Portability and Accountability Act (HIPAA).

## 4. Digital Twins in Precision Nutrition

The intensifying utilization of digital twins across sectors is powering its rise finally in healthcare [15]. They allow physical objects, processes, policies, and persons and their behaviors to be virtually represented across numerous different assumptions, conditions, and changes throughout their development and deployment lifecycle. Doing so allows a more cost-efficient design and deployment of the products, processes, and policies without the traditional slow and expensive approach of trial-and-error in the ‘real world’. Consider operating on a three-dimensional model of a patient’s complex heart to get the surgical approach right first, instead of going in ‘blind’ and doing the real thing on a real patient without robust preparation. Digital twins have recently been supercharged by AI applied to Big Data, especially through the Internet of Things (IoTs) spanning the global network of inter-connected ‘smart objects’, such as smart phones communicating with each other and the cloud (remote servers managing data, applications, and services). This is because smarter AI running on larger data enables more rapid, affordable, and robust digital twins, which is particularly beneficial for countries, companies, and healthcare organizations with less resources for slower and more expensive development lifecycles. When digital twin technologies first were born out of necessity in the 1960s by the United States NASA (National Aeronautics and Space Administration), they were used to rapidly and successfully model on earth the Apollo 13 spacecraft that had suffered a catastrophic accident in space, and thus generate the now famous detailed procedure broadcasted remotely to the astronauts on how to modify their lunar module to return them safely home. Flashforward to 2024 after decades of continuous digital twin evolution and digital technologies on which they are computed, and there are growing applications in healthcare with ‘mirror twins’ (in which twin models have dynamic behavior such as with intelligent randomized control trials and in silico clinical trials modeling actual patients’ response to different treatments before running the trials in vivo), ‘shadow twins’ (in which models regularly incorporate new data such as in medical device design), and the most advanced form of ‘intelligent twins’ (in which self-adaptive models upgraded by AI can automatically utilize new data to modify themselves and even communicate with other twins). Accordingly, healthcare digital twins are also moving into nutrition with case uses already showing personalized diet plans for individual health needs and preferences to encourage higher compliance, informed by the precision nutrition approach which adapts clinical nutrition support based on individual clinical, socioeconomic, psychosocial, diet, metabolic, microbiome, and genetic data [16]. Instead of telling patients to abide by general national guidelines through a slow trial-and-error period with high drop out and noncompliance, such digital twins can help rapidly model the various combinations of which foods people need, can afford, and want to consume through digital twins of themselves, while updating this guidance especially with the more advanced intelligent digital twins in response to early health results of how these dietary plans are working. Such innovations already seem to be bearing fruit. By 2023, an Indian–American precision nutrition randomized trial of 319 subjects with diabetes showed that AI-generated digital twins compared to the standard of care enabled more personalized diet plans with a resultant nearly 10-fold improved HbA1c improvement, a key target in ASCVD prevention and management [17]. Nonetheless, there are key persistent challenges to implementing digital twins for such precision nutrition, which include accessibility (to practitioners able to create them), identifiability (of which physical objects can be appropriately modeled), robustness (of models especially when there is limited prior data to guide their creation), errors (in which model results deviate from ultimate real-world outcomes), and trustworthiness (understanding to what extent digital twins can be relied on to guide real-world decisions versus only inform further R&D).

## 5. AI-Enabled Culinary Medicine as Medical Education and Treatment

A 2019 *Lancet* systematic review of 24 studies from Africa, the Middle East, Australasia, Europe, and the United States documented the widespread deficits of nutrition training in medical education which undermines future physicians’ competence and confidence in counseling patients in nutrition [18]. Though obesity in America is approaching half of the population, approximately 75% of its medical schools do not reach the minimum recommended hours of nutrition education, while over two-thirds of physicians fail to provide dietary counseling to the majority of their patients despite most having ASCVD or its risk factors [19]. A 2022 meta-analysis of nutrition education spanning 1698 medical trainees demonstrated that the highest quality study and top performing intervention was by an AI-augmented hands-on cooking and nutrition education curriculum in culinary medicine which led to the greatest boost in trainee competency providing patients nutrition education. This physician-supervised and Mediterranean diet (MedDiet)-based curriculum was first taught by dieticians and chefs to medical students then resident physicians then attending physicians. Those healthcare workers then provided the curriculum to patient communities, with the MedDiet chosen compared to other diets given its widespread utilization and generally accepted morbidity and mortality benefits, especially for ASCVD [20,21]. Among 5847 subjects across 45 universities and hospitals, the odds of high and medium versus low MedDiet diet adherence was significantly increased with this curriculum compared to the standard of care by over 10-fold for resident physicians, almost 10-fold for medical students (a nearly 10-fold improvement compared to the initial year of program creation), over 5-fold for practicing physicians, and nearly 3-fold for patients. A randomized trial of 41 families particularly from lower-income communities with limited healthy food offerings showed that compared to traditional dietary counseling, this culinary medicine curriculum was associated with nearly three-times increased odds of MedDiet adherence, while also significantly reducing their weekly food costs [22]. The preceding prospective cohort study across 20 medical schools and 3248 medical trainees showed that this curriculum also more than doubled the odds of trainee competencies providing patients with nutrition education and halved their odds of sugary soft drink consumption [23]. The seminal Bayesian adaptive pilot randomized trial concurrently showed that this curriculum taught by medical trainees compared to the standard of care improved patients’ HbA1c, blood pressure, and cholesterol in the first such known randomized trial of culinary medicine [24]. As the Principal Investigator, trial designer, and senior data scientist for the preventive cardiology cohort study within which the above sub-studies were published—Cooking for Health Optimization with Patients (CHOP; National Library of Medicine ClinicalTrials.gov ID NCT03443635)—I (DJM) sought to anchor continuous program improvement on the AI method of machine learning to inform near real-time insights for personalizing and scaling the education intervention across the national network of partners targeting ASCVD risk factors and diseases to establish the evidence-based foundation for culinary medicine as medical education and treatment, with particular focus on lower-income underserved communities. This methodology has been fine-tuned into the comprehensive end-to-end technique of AI-driven Computational Ethics and policy analysis (AiCE), which applies Quantum Bayesian Machine learning-augmented Propensity Score translational (QBAM-PS) statistics for clinical causal inference, cost-effectiveness, and human-centric Personalist Social Contract ethics that runs on advanced deep learning software and powered by hybrid classical–quantum computing platforms [4]. AiCE thus may provide a responsible, trustworthy, reliable, and comprehensive platform for the continuous optimization of healthy diet for ASCVD interventions (as though there is a growing awareness of the need for ethical and sustainability guardrails in AI-augmented nutrition interventions, there are currently no other known comprehensive methodologies uniting the diverse dimensions above for efficient and equitable interventions in real-world practice for the pluralistic and complex global health ecosystem) [25,26]. Culinary medicine has since continued to grow as a more clinically, financially, and ecologically sustainable medical education model, as patients longitudinally improving their healthy diet behaviors can reduce their odds of ASCVD incidence, progression, and acute exacerbations requiring more financially and ecologically expensive treatments. After I (DJM) published the first known quantitative study of culinary medicine in 2014, there has been a steady rise to over 149 related publications internationally with diverse partners expanding and adopting the related curriculum for their community needs (including for future and practicing dieticians, chefs, nurses, and physicians). The culinary medicine curriculum has additionally been translated into a multi-phase study by the National Institutes of Health for minorities in an urban food desert [27]. It has also been codified within the American College of Culinary Medicine with its ‘Health meets Food’ curriculum (licensed to over 65 medical schools, residency programs, and nursing schools by 2024) and related continuing medical education or CME credits provided to over 20,000 attendees and its Certified Culinary Medicine Specialist (CCMS) [28]. It has also been increasingly synced with clinical workflows including through electronic consultations or eConsults in culinary medicine for personalized nutrition recommendations through electronic health record systems by CCMS clinicians [29].

## 6. Conclusions

This novel narrative review seeks to provide the first known overview of the state-of-the-art in clinical interventions and public health policies in healthy diets for ASCVD, accelerated by health equity-focused AI, and written from the first-hand practitioner perspective in the last explosive decade of this evolution. The review summarizes its emerging trends and leading use cases in population health risk stratification and precision public health, AI democratizing clinical diagnosis, digital twins in precision nutrition, and AI-enabled culinary medicine as medical education and treatment. Efforts are underway by a growing number of teams globally to publish the empirical analyses of the related case uses to advance the evidence-based foundation for more clinically effective, financially efficient, and societally equitable dietary and nutrition interventions for ASCVD.

The biological relationship between ASCVD and poor diet is already well understood (along with its massive societal cost), as are the policy recommendations for them are well codified [4]. Yet, AI’s growing applications for ASCVD and diet are upending the status quo by offering exciting new promises, along with sobering new challenges and cautions. This review has surveyed such related cases which taken together can be understood as delivering more efficient successes but with smarter but riskier technologies that we have limited experience regulating, using, and fixing. There is a seemingly inexhaustible historic and multi-sector growth of AI, fueled by ever-increasing data and the profit these technologies enable through the cheaper optimization of R&D and processes. Yet, there is also decentralized and non-linear technological development, which is consistently faster than government regulation and population adjustment (with growing numbers of stakeholders creating and applying them even with constrained resources, while workforces and even cultures face unpredictable changes from this industrial revolution of dynamic automated intelligence at global scale). Such challenges are exacerbated by healthcare being the last sector to be disrupted by AI, while it alone holds nearly one-third of the world’s data, which is also among the most sensitive. Therefore, the societal implications of AI generally (and its specific role for our purposes here of accelerating the dietary prevention of ASCVD) must be considered not only by its creators but also its practitioners. This is particularly acute for us as healthcare providers who have a unique duty to the good of our patients and the equity for our communities, directly touching one of the core dimensions of who we are as people—our wellbeing, and the sensitive data related to it. The typically recognized societal implications for this technology and its health applications above include its indefensible biases, accountability, privacy, security, and workforce disruption, while the under-reported implications include cultural imperialism, exploitation, and technical reductionism (of persons simply to consumers). Arguably, the most influential paradigm of policy recommendations to address them is that which is institutionalized in the European AI Act as a state-backed, rights-based, multilateral approach to understanding AI on a spectrum of risks (in which low-risk applications should have less regulation if any and higher risk applications should have more regulation and in some cases even outran bans). Given how the European Union is the world’s largest trading bloc and market, AI companies and health partners are, therefore, increasingly globally scaling their products and services to have this European compliance-by-design. Anu Bradford describes this ‘Brussels Effect’ as the continent’s unilateral regulatory globalization making its paradigm the de facto one for us all. Practically for dietary approaches to ASCVD as a later-stage adopter of such technologies, initial policy recommendations, therefore, seem to be following the lead of the rest of the healthcare sector to AI. Accordingly, this field seems to be adopting this type of regulatory and related application paradigm: use the least amount of approved data required to develop and deploy the most effective, affordable, and fair technologies in mutually beneficial, transparent, and accountable partnerships among AI creators, healthcare workers, and patients.

Aside from such social implications, the promise and caution of such early AI applications in this dietary approach to ASCVD highlight specific future research directions to advance a defensible and continually improved development for them. These include a more granular and individualized biological understanding of how specific diets impact specific patients using multi-omics (genomics, epigenomics, transcriptomics, proteomics, and metabolomics). This can then inform research in precision medicine and precision public health to apply the ever-moving state-of-the-art in this field to specific patients and populations to ensure the right treatment is given at the right time and right place to the right people. Yet, expanded economic research is also required to ensure the R&D, interventions, and scale-up for them are accessible and affordable, especially for those who most need it in communities and countries with the least resources for them. And finally, broader and deeper ethical research is required to understand the multicultural, interreligious, and pluralistic perspectives of diverse people, peoples, and belief systems in this field and how substantive and agile ethical convergence can be achieved, sustained, and institutionalized as AI technologies and their applications in dietary approaches to ASCVD change. Integrated research methodologies such as AiCE above may be a helpful paradigm to address these different dimensions for a global audience while embedding effective products produced from it in real-world workflows, but rooted and focused on the individual person this field is meant to serve. There is an ever-growing body of recent literature that offers complementary advances toward this goal as well. These include efforts with a particular diet focused (given its biological centrality) on steadily improving ASCVD prevention with AI, including improved risk assessment, screening, guideline adherence, and real-world integration with clinical workflows and public health programs [4,11,30,31,32,33,34,35,36,37,38,39,40]. Catalyzing such health advances include the technical advances in blockchain and swarm learning for data security, classical–quantum computing hybrid platforms for greater computing power, and rights-based collaboration and managed strategic competition of sovereign AI. Considering the practical hope springing up from this review’s use cases and the above research directions (tempered by appropriate caution), is it, therefore, ultimately possible that the more sophisticated the interventions, the smarter the analyses, and the more sustainably impactful such collective health solutions are, the better we are able to do the simple but necessary thing of returning to the dinner table together as the global human family. There can we better share the fruits of our international efforts for the local benefit of all patients and communities (who share the common risks and costs of ASCVD, and the benefit of and savings from the responsible use of technologies for it).

## References

[1] Sikand G., Severson T. (2020). Top 10 dietary strategies for atherosclerotic cardiovascular risk reduction. Am. J. Prev. Cardiol..

[2] World Bank DataBank. Food Prices for Nutrition. https://www.worldbank.org/en/programs/icp/brief/foodpricesfornutrition.

[3] Food and Agriculture Organization The State of Food and Agriculture. https://openknowledge.fao.org/server/api/core/bitstreams/8a80e31b-3c41-419d-a11d-0b62e4b2528a/content/state-of-food-and-agriculture-2023/executive-summary.html.

[4] Monlezun D.J. (2024). Responsible Article Intelligence Re-Engineering the Global Public Health Ecosystem.

[5] Bankhwal M., Chiu M., Bisht A., Roberts R., van Heteren A. AI for Social Good: Improving Lives and Protecting the Planet. McKinsey & Company. https://www.mckinsey.com/capabilities/quantumblack/our-insights/ai-for-social-good.

[6] Cai C., Li H., Zhang L., Li J., Duan S., Fang Z., Li C., Chen H., Alharbi M., Ye L. (2024). Machine learning identification of nutrient intake variations across age groups in metabolic syndrome and healthy populations. Nutrients.

[7] Jordan G., Ridder D., Joost S., Vollenweider P., Preisig M., Marques-Vidal P., Guessous I., Vaucher J. (2024). Spatial analysis of 10-year predicted risk and incident atherosclerotic cardiovascular disease: The CoLaus cohort. Sci. Rep..

[8] Christakis N.A., Fowler J.H. (2007). The spread of obesity in a large social network over 32 years. N. Engl. J. Med..

[9] Dalakoti M., Wong S., Lee W., Lee J., Yang H., Loong S., Loh P.H., Tyebally S., Djohan A., Ong J. (2024). Incorporating AI into cardiovascular diseases prevention-insights from Singapore. Lancet Reg. Health-West. Pac..

[10] World Health Organization The Global Health Observatory. Global Health Workforce Statistics Database. https://www.who.int/data/gho/data/themes/topics/health-workforce.

[11] Awasthi S., Sachdeva N., Gupta Y., Anto A.G., Asfahan S., Abbou R., Bade S., Sood S., Hegstrom L., Vellanki N. (2023). Identification and risk stratification of coronary disease by artificial intelligence-enabled ECG. eClinicalMedicine.

[12] Monlezun D.J., Iliescu G.D., Ali A., Khalaf S., Javaid A., Kim J.W., Iliescu C. Dietary counseling disparities, mortality, and cost in coronary artery disease, obesity, and cancer in 47,900 hospitalizations. Proceedings of the Multinational Association of Supportive Care in Cancer Annual Conference.

[13] Jalepalli S.K., Gupta P., Dekker A.L.A.J., Bermejo I., Kar S. (2024). Development and validation of multicentre study on novel Artificial Intelligence-based Cardiovascular Risk Score (AICVD). Fam. Med. Community Health.

[14] Biddappa R. Reliance and NVIDIA Partner to Advance AI in India, for India. NVIDIA. https://nvidianews.nvidia.com/news/reliance-and-nvidia-partner-to-advance-ai-in-india-for-india.

[15] Katsoulakis E., Wang Q., Wu H., Shahriyari L., Fletcher R., Liu J. (2024). Digital twins for health: A scoping review. NPJ Digit. Med..

[16] Vahdati M., Saghiri A.M., HamlAbadi K.G., El Saddik A. (2023). Digital twins for nutrition. Digital Twins for Healthcare.

[17] Joshi S., Shamanna P., Dharmalingam M., Vadavi A., Keshavamurthy A., Shah L. (2023). Digital twin-enabled personalized nutrition improves metabolic dysfunction-associated fatty liver disease in type 2 diabetes: Results of a 1-year randomized controlled study. Endocr. Pract..

[18] Crowley J., Ball L., Hiddink G.J. (2019). Nutrition in medical education: A systematic review. Lancet Planet Health.

[19] Monlezun D.J., Carr C., Niu T., Nordio F., DeValle N., Sarris L., Harlan T. (2022). Meta-analysis and machine learning-augmented mixed effects cohort analysis of improved diets among 5847 medical trainees, providers and patients. Public Health Nutr..

[20] Estruch R., Ros E., Salas-Salvadó J., Covas M.I., Corella D., Arós F., Gómez-Gracia E., Ruiz-Gutiérrez V., Fiol M., Lapetra J. (2018). Primary prevention of cardiovascular disease with a mediterranean diet supplemented with extra-virgin olive oil or nuts. N. Engl. J. Med..

[21] Fan H., Wang Y., Ren Z., Liu X., Zhao J., Yuan Y., Fei X., Song X., Wang F., Liang B. (2023). Mediterranean diet lowers all-cause and cardiovascular mortality for patients with metabolic syndrome. Diabetol. Metab. Syndr..

[22] Razavi A.C., Sapin A., Monlezun D.J., McCormack I.G., Latoff A., Pedroza K., McCullough C., Sarris L., Schlag E., Dyer A. (2021). Effect of culinary education curriculum on Mediterranean diet adherence and food cost savings in families: A randomised controlled trial. Public Health Nutr..

[23] Monlezun D.J., Dart L., Vanbeber A., Smith-Barbaro P., Costilla V., Samuel C., Terregino C.A., Abali E.E., Dollinger B., Baumgartner N. (2018). Machine learning-augmented propensity score-adjusted multilevel mixed effects panel analysis of hands-on cooking and nutrition education versus traditional curriculum for medical students as preventive cardiology: Multisite cohort study of 3,248 trainees over 5 years. Biomed. Res. Int..

[24] Monlezun D.J., Kasprowicz E., Tosh K.W., Nix J., Urday P., Tice D., Sarris L., Harlan T.S. (2015). Medical school-based teaching kitchen improves HbA1c, blood pressure, and cholesterol for patients with type 2 diabetes: Results from a novel randomized controlled trial. Diabetes Res. Clin. Pract..

[25] Namkhah Z., Fatemi S.F., Mansoori A., Nosratabadi S., Ghayour-Mobarhan M., Sobhani S.R. (2023). Advancing sustainability in the food and nutrition system: A review of artificial intelligence applications. Front. Nutr..

[26] Detopoulou P., Volugaridou G., Moschos P., Giaginis C., Panoutsopoulous G.I., Papadopoulou S.K. (2023). Artificial intelligence, nutrition, and ethical issues: A mini-review. Clin. Nutr. Open Sci..

[27] Farmer N., Powell-Wiley T.M., Middleton K.R., Roberson B., Flynn S., Brooks A.T., Kazmi N., Mitchell V., Collins B., Hingst R. (2020). A community feasibility study of a cooking behavior intervention in African-American adults at risk for cardiovascular disease: DC COOKS (DC Community Organizing for Optimal culinary Knowledge Study) with Heart. Pilot Feasibility Stud..

[28] The American College of Culinary Medicine Health Meets Food. Continuing Medical Education. https://culinarymedicine.org/certified-culinary-medicine-specialist-program/apply/.

[29] Albin J.L., Mignucci A.J., Siler M., Dungan D., Neff C., Faris B., McCardell C.S., Harlan T.S. (2024). From clinic to kitchen to electronic health record: The background and process of building a culinary medicine eConsult service. J. Multidiscip. Health.

[30] Van den Eynde J., Lachmann M., Laugwitz K.L., Manlhiot C., Kutty S. (2023). Successfully implemented artificial intelligence and machine learning applications in cardiology: State-of-the-art review. Trends Cardiovasc. Med..

[31] Eng D., Chute C., Khandwala N., Rajpurkar P., Long J., Shleifer S., Khalaf M.H., Sandhu A.T., Rodriguez F., Maron D.J. (2021). Automated coronary calcium scoring using deep learning with multicenter external validation. NPJ Digit. Med..

[32] Boonstra M.J., Weissenbacher D., Moore J.H., Gonzalez-Hernandez G., Asselbergs F.W. (2024). Artificial intelligence: Revolutionizing cardiology with large language models. Eur. Heart J..

[33] Sarraju A., Bruemmer D., Van Iterson E., Cho L., Rodriguez F., Laffin L. (2023). Appropriateness of Cardiovascular Disease Prevention Recommendations Obtained From a Popular Online Chat-Based Artificial Intelligence Model. JAMA.

[34] Abdelrahman K., Shiyovich A., Huck D.M., Berman A.N., Weber B., Gupta S., Cardoso R., Blankstein R. (2024). Artificial Intelligence in Coronary Artery Calcium Scoring Detection and Quantification. Diagnostics.

[35] Ihdayhid A.R., Lan N.S.R., Williams M., Newby D., Flack J., Kwok S., Joyner J., Gera S., Dembo L., Adler B. (2023). Evaluation of an artificial intelligence coronary artery calcium scoring model from computed tomography. Eur. Radiol..

[36] Krittanawong C., Rogers A.J., Aydar M., Choi E., Johnson K.W., Wang Z., Narayan S.M. (2020). Integrating blockchain technology with artificial intelligence for cardiovascular medicine. Nat. Rev. Cardiol..

[37] Somani S., van Buchem M.M., Sarraju A., Hernandez-Boussard T., Rodriguez F. (2023). Artificial Intelligence-Enabled Analysis of Statin-Related Topics and Sentiments on Social Media. JAMA Netw. Open.

[38] Sarraju A., Coquet J., Zammit A., Chan A., Ngo S., Hernandez-Boussard T., Rodriguez F. (2022). Using deep learning-based natural language processing to identify reasons for statin nonuse in patients with atherosclerotic cardiovascular disease. Commun. Med..

[39] Miller RJ H., Pieszko K., Shanbhag A., Feher A., Lemley M., Killekar A., Kavanagh P.B., Van Kriekinge S.D., Liang J.X., Huang C. (2023). Deep Learning Coronary Artery Calcium Scores from SPECT/CT Attenuation Maps Improve Prediction of Major Adverse Cardiac Events. J. Nucl. Med. Off. Publ. Soc. Nucl. Med..

[40] Zeleznik R., Foldyna B., Eslami P., Weiss J., Alexander I., Taron J., Parmar C., Alvi R.M., Banerji D., Uno M. (2021). Deep convolutional neural networks to predict cardiovascular risk from computed tomography. Nat. Commun..

